# Petra Terhoeven und Dirk Schumann 2021: *Strategien der Selbstbehauptung. Vergangenheitspolitische Kommunikation an der Universität Göttingen (1945–1965) (= Veröffentlichungen des Zeitgeschichtlichen Arbeitskreises Niedersachsen, Bd. 36)* und Christa Klein 2020: *Elite und Krise. Expansion und „Selbstbehauptung“ der Philosophischen Fakultät Freiburg 1945–1967 (= Wissenschaftskulturen. Reihe III: Pallas Athene, Bd. 54)*

**DOI:** 10.1007/s00048-021-00320-9

**Published:** 2021-12-21

**Authors:** Alexander Mayer

**Affiliations:** Universität der Bundeswehr München, Neubiberg, Deutschland

**Petra Terhoeven und Dirk Schumann 2021:* Strategien der Selbstbehauptung. Vergangenheitspolitische Kommunikation an der Universität Göttingen (1945–1965) (= Veröffentlichungen des Zeitgeschichtlichen Arbeitskreises Niedersachsen, Bd. 36).*** Göttingen: Wallstein, geb., 357 S., 7 Abb., 34,00 €, ISBN: 978-3-8353-3836‑4.

**Christa Klein 2020:** ***Elite und Krise. Expansion und „Selbstbehauptung“ der Philosophischen Fakultät Freiburg 1945–1967 (= Wissenschaftskulturen. Reihe III: Pallas Athene, Bd. 54).*** Stuttgart: Steiner, geb., 394 S., 22 Abb., 66,00 €, ISBN: 978-3-515-12599‑4.

Seit den 1990er Jahren hat die wissenschaftshistorische Forschung gezeigt, in welchem Maß deutsche Wissenschaftler unter dem NS-Regime zur Selbstmobilisierung bereit waren. Bekannt ist auch, dass nach dem Krieg die überwiegende Zahl derer, die sich in den Dienst der nationalsozialistischen Politik gestellt hatten, ihre Posten behielt oder zumindest nach wenigen Jahren wieder auf eine Professur gelangte, während nur eine kleine Minderheit ideologisch besonders exponierter Wissenschaftler dauerhaft ausgeschlossen blieb. Die Monografie von Christa Klein sowie der von Petra Terhoeven und Dirk Schumann herausgegebene Sammelband befassen sich mit dieser „Selbstbehauptung“ wissenschaftlicher Eliten nach 1945. Beide Publikationen nehmen die kommunikativen Strategien in den Blick, mittels derer es deutschen Professoren gelang, nicht nur ihre institutionelle Position, sondern auch ihre Autorität und Deutungsmacht zu sichern.

Der Sammelband *Strategien der Selbstbehauptung* rückt dabei, wie Petra Terhoeven formuliert, die Suche der Wissenschaftler nach „gesellschaftlich beglaubigten Sagbarkeitsregeln“ in den Fokus, „mit Hilfe derer die einzelnen beteiligten Akteure ihre Vergangenheit überschreiben und sich innerhalb veränderter Bedeutungshierarchien erfolgreich neupositionieren konnten“ (11). Die einzelnen Beiträge verfolgen überwiegend einen akteurszentrierten Ansatz. Der Zusammenhang zwischen öffentlicher Kommunikation über die nationalsozialistische Vergangenheit und dem Streben nach individueller Rehabilitation wird besonders an den Beispielen der Historiker Percy Ernst Schramm, Walther Hubatsch und Hermann Heimpel deutlich. So zeigt Kerstin Thieler, wie sich der Mittelalterhistoriker Schramm, der von 1943 bis 1945 das Kriegstagebuch des Obersten Heereskommandos führte, in der westdeutschen Öffentlichkeit als Aufklärer und Experte für den Nationalsozialismus und den Zweiten Weltkrieg profilierte und damit seine Nähe zum NS-Regime mit dem Gestus wissenschaftlicher Objektivität überdeckte.

Die Beiträge des Bandes stellen aber nicht allein die NS-belasteten Wissenschaftler in den Fokus, sondern thematisieren am Beispiel der Physiker James Franck und Max Born sowie dessen Ehefrau Hedwig Born auch das Verhalten von Emigrierten. Dabei wird deutlich, wie schwierig deren Positionierung angesichts von Vereinnahmungsversuchen, verweigerter Wiedergutmachung und mangelnder Selbstkritik seitens ihrer in Deutschland verbliebenen „Kollegen“ war. Emigrierte Wissenschaftler wurden schon dadurch ausgegrenzt, dass sie ihre während der NS-Zeit neu besetzten Stellen nicht zurückerhielten. Das Beispiel der Nobelpreisträger James Franck und Max Born ist dabei nicht unbedingt repräsentativ, da sich die beiden prominenten Wissenschaftler, wie Kerstin Thieler und Jan Renken schildern, mit Versöhnungsinszenierungen konfrontiert sahen – zum Beispiel der Verleihung der Ehrenbürgerschaft der Stadt Göttingen –, deren Zweck darin bestand, jene zu rehabilitieren, die sich mit dem Regime arrangiert hatten. Renken zeichnet zudem nach, wie die persönliche Erfahrung mangelnder Bereitschaft zur Wiedergutmachung sowie die allgemeine Sorge um das politische Klima in der Bundesrepublik das Ehepaar Max und Hedwig Born zu öffentlichen erinnerungspolitischen Interventionen veranlasste. Hier wie auch im Fall von Ehrengard Schramm, die dem NS-Regime deutlich kritischer gegenübergestanden hatte als ihr Ehemann, sich aber gerade deshalb für seine Rehabilitation einsetzen konnte, tritt auch die Rolle der oft ausgeblendeten Professorengattinnen hervor.

Der Band zeichnet ein detailreiches Bild insbesondere der privaten vergangenheitspolitischen Kommunikation, das sich der Auswertung der Nachlässe der zentralen Akteure verdankt. So sehr der von Terhoeven in der Einleitung formulierte Ansatz überzeugt, lassen die einzelnen Beiträge aber eine konsequentere Anwendung des diskursanalytischen Instrumentariums, wie sie der Begriff der „Sagbarkeitsregeln“ nahelegt, vermissen. Solche Regeln werden bisweilen eher konstatiert als in ihrer Genese und Reproduktion erklärt. Öfter würde man sich Überlegungen zu den „Machtasymmetrien innerhalb der Erinnerungskultur“ wünschen, wie sie Renken in seinem Beitrag zu Max und Hedwig Born anstellt (266). Es besteht zudem ein gewisser Konflikt zwischen dem systematischen, auf gesellschaftlich verbindliche Sagbarkeitsregeln gerichteten Erkenntnisinteresse und der personenzentrierten Herangehensweise in den Einzelstudien.

Christa Klein geht es in ihrer Monografie weniger um die persönliche Vergangenheitsbewältigung ihrer Akteure als um die Strategien, mittels derer geisteswissenschaftliche Professoren ihre elitäre Position in der Universität sowie ihre Deutungsmacht in der Öffentlichkeit zu behaupten suchten. Eine entscheidende Rolle schreibt sie dabei der „idealistischen Krisenrhetorik“ zu, als deren Trägergruppe in Freiburg sie eine „Krisengeneration“ von Professoren identifiziert, die überwiegend zwischen 1937 und 1951 berufen wurden. Diese Professoren verbindet, dass sie „mit einem allgegenwärtigen Krisendiskurs wissenschaftlich sozialisiert worden waren, den sie in der Zweiten Nachkriegszeit wieder aufnahmen und für sich zu nutzen verstanden“ (195). Drei Dimensionen dieses Krisendiskurses hebt Klein hervor – nämlich die Beschwörung einer deutschen Universitätsidee, die Ablehnung von Akademisierungsprozessen und „Überfüllung“ sowie ein gegen „Positivismus“ und disziplinäre Ausdifferenzierung gerichtetes idealistisch-holistisches Wissenschaftsverständnis. Professoren wie die Historiker Gerhard Ritter und Gerd Tellenbach griffen damit auf kulturkritische Diskurse aus der ersten Hälfte des 20. Jahrhunderts zurück, die zum Teil auch im Nationalsozialismus fortgeschrieben worden waren, nutzten diese aber nun, um die NS-Belastung der akademischen Elite zu übertünchen. Eine modernitätskritische Deutung des Nationalsozialismus als Produkt von Säkularisierung und Orientierungsverlust in der „Massengesellschaft“ diente dazu, die auf Basis eines idealistisch-holistischen Wissenschaftsverständnisses postulierte gesellschaftliche Orientierungsfunktion der Geisteswissenschaften zu begründen.

Konfrontiert mit einem zunehmenden Modernisierungsdruck entwickelte sich die Krisenrhetorik in den 1950er Jahren „zu einer filternden Integrationsstrategie, die vorwiegend die Elite stärkende Modernisierungsaspekte durchließ“ (299). Sowohl universitätsgeschichtlich als auch mit Blick auf aktuelle Debatten über universitäre Personalstrukturen besonders interessant ist die von Klein detailliert nachgezeichnete Reaktion auf die rapide steigenden Studierendenzahlen. Letztlich wurde die „Überfüllungskrise“ in erster Linie durch einen überproportionalen Zuwachs des Mittelbaus und dort wiederum Stellen für Nicht-Habilitierte bewältigt. Dafür, dass sich diese auf hierarchischer Aufgabenteilung basierende Lösung durchsetzte und nicht etwa das zeitgenössisch diskutierte Modell der Studienprofessur oder ein stärkerer Ausbau der Professuren, macht Klein (neben Kostenerwägungen) das im Modus der Krisenrhetorik artikulierte elitäre Selbstverständnis der Professoren verantwortlich.

Das sich auf Humboldt berufende Selbstverständnis der Krisengeneration entsprach allerdings – wie Klein mit Fokus auf Tellenbach und den Politikwissenschaftler Arnold Bergstraesser argumentiert – immer weniger der realen Praxis. Beide verkörperten den „Professorentypus des Wissenschaftsmanagers […], dessen Hauptaufgabe nicht in der praktizierten Forschung und Lehre bestand, sondern in deren Organisation und Koordination, Finanzakquise, Nachwuchsförderung, Lobby- und Netzwerkarbeit“ und der nun zum „hegemonialen Typus“ aufstieg (207).

Die Stärke dieser Arbeit liegt in der Verknüpfung von Institutionen- und Ideengeschichte, wobei es Klein gelingt, die konkreten Interessen hinter der Krisenrhetorik aufzudecken, deren Leerstellen zu benennen und Inkonsistenzen zwischen der Rhetorik und der wissenschaftlichen beziehungsweise wissenschaftsorganisatorischen Praxis der Professoren aufzuzeigen. Es drängt sich allerdings die Frage auf, welchen Einfluss die Entwicklungen in anderen Disziplinen auf den Wandel der universitären Personalstrukturen und den Aufbau der Studiengänge hatten und wie wirkmächtig die beschriebene Krisenrhetorik jenseits des geisteswissenschaftlichen Feldes war. Aber dies weist bereits über die mit guten Gründen gezogenen Grenzen dieser Studie hinaus.

